# Proteomics identifies differentially expressed proteins in neonatal murine thymus compared with adults

**DOI:** 10.1186/1477-5956-10-65

**Published:** 2012-11-08

**Authors:** Xinze Cai, Wenyue Huang, Ying Qiao, Yang Chen, Shuyan Du, Dong Chen, Shuang Yu, Ruichao Che, Yi Jiang

**Affiliations:** 1 Central Laboratory, First Affiliated Hospital of China Medical University, Shenyang, 110001, China; 2 Department of Immunology, College of Basic Medical Sciences, China Medical University, Shenyang, 110001, China; 3 Department of Biotherapy, Fourth Affiliated Hospital of China Medical University, Shenyang, 110032, China; 4 Department of Dermatology, First Affiliated Hospital of China Medical University, Shenyang, 110001, China

**Keywords:** DIGE, Thymus, Development, Proteomics, Immune

## Abstract

**Background:**

The thymus is an immune organ essential for life and plays a crucial role in the development of T cells. It undergoes a fetal to adult developmental maturation process occurring in mouse during the postnatal months. The molecular modifications underlying these ontogenic changes are essentially unknown. Here we used a differential proteomic-based technique (2D-Difference Gel Electrophoresis) coupled with matrix-assisted laser desorption/ionization-time of flight (MALDI-TOF) mass spectrometry to search for key proteins in the postnatal development of the thymus. Eight different BALB/c mice were used in the study: four mice aged of 1 day (neonatal) and four mice aged of 60 days (adult). Protein samples derived from thymus were labeled and run in 2D-PAGE (Two-Dimensional Polyacrylamide Gel Electrophoresis). One whole-thymus tissue from each mouse was run on gels and each gel containing a pooled sample of the eight mice was run in parallel. The pooled sample was set as the internal pool, containing equal amount of each protein extract used in the experiment. Gels were matched and compared with Difference In-gel Analysis software. Differential spots were picked, in-gel digested and peptide mass fingerprints were obtained.

**Results:**

Among the differentially regulated proteins in neonatal thymus group, 111 proteins were identified by mass spectrometry, of which 95 proteins were up-regulated and 16 proteins were down-regulated. The identified proteins belong to several functional categories, including cell proliferation, cycle and apoptosis, transcription regulation, signal transduction, nucleotide processing, proteolysis and translation, protein folding, metabolism, oxidoreduction, cytoskeleton, immune response, and embryonic development. The major interaction networks comprised of cellular function and maintenance, cellular assembly and organization, and metabolism were also identified by STRING analysis.

**Conclusions:**

The demonstrated molecular changes are relevant for understanding thymus development as well as neonatal immune function, and they provide the diagnostic disease markers. Further studies will be required to describe in detail the role of the identified proteins in thymus maturation and in the specific functions of neonatal thymus.

## Introduction

The thymus is essentially an epithelial organ, containing many developing lymphocytes and playing a crucial role in the development of T cells. Histologically, the thymus can be broadly divided into two subcompartments, the cortex and the medulla, each of which contains distinct populations of thymic epithelial cells (TECs) and mesenchymal cells, endothelial cells and dendritic cells [1]. The microenvironment of the thymus can produce a diverse repertoire of peripheral T cells, and the correct patterning and organization of thymus stromal components are crucial for thymus function. Defects in thymus function can result in serious health consequences, including immunodeficiency or autoimmunity.

Thymus undergoes major homeostatic postnatal functional modifications and the underlying molecular mechanisms are essentially unknown. In recent years, much progress has been made in identifying the transcription factors and signaling pathways that play a role in thymus organogenesis and T cell development [2]. Although much of these molecular insights involved in development and immune reactions come from gene expression data analyzed by microarray technologies, they are unable to provide information concerning translational regulation of expression or post-translational modification [3].

Proteomic analysis of global changes in protein expressed in neonatal and adult murine organs provides a useful method for detecting proteins that play a role in the developing processes [4-6]. It is proposed that during the development of the thymus, various signals are present in neonatal thymus which differ from the adult thymus. The homeostasis of neonatal thymus microenvironment is critical for the metabolism and immune response [7]. Systematic analysis of thymus protein expression profiles including information about protein signatures, localization and their quantitative changes are thus useful to thymus development and maturation.

Recently the two-dimensional difference gel electrophoresis (2-D DIGE) technique with fluorescent dyes has allowed quantitative analysis of separated proteins with high sensitivity [8]. In this study, we performed a comparative proteomic analysis of differentially expressed proteins in the thymus of mice aged 1 day (neonates) and 60 days (adults), and the function analysis and the crosstalk of the proteins would be provided, aiming to search for key proteins in the postnatal development of the thymus.

## Results

### Identification of differentially expressed proteins in murine thymus using 2-DE

**Figure 1:**
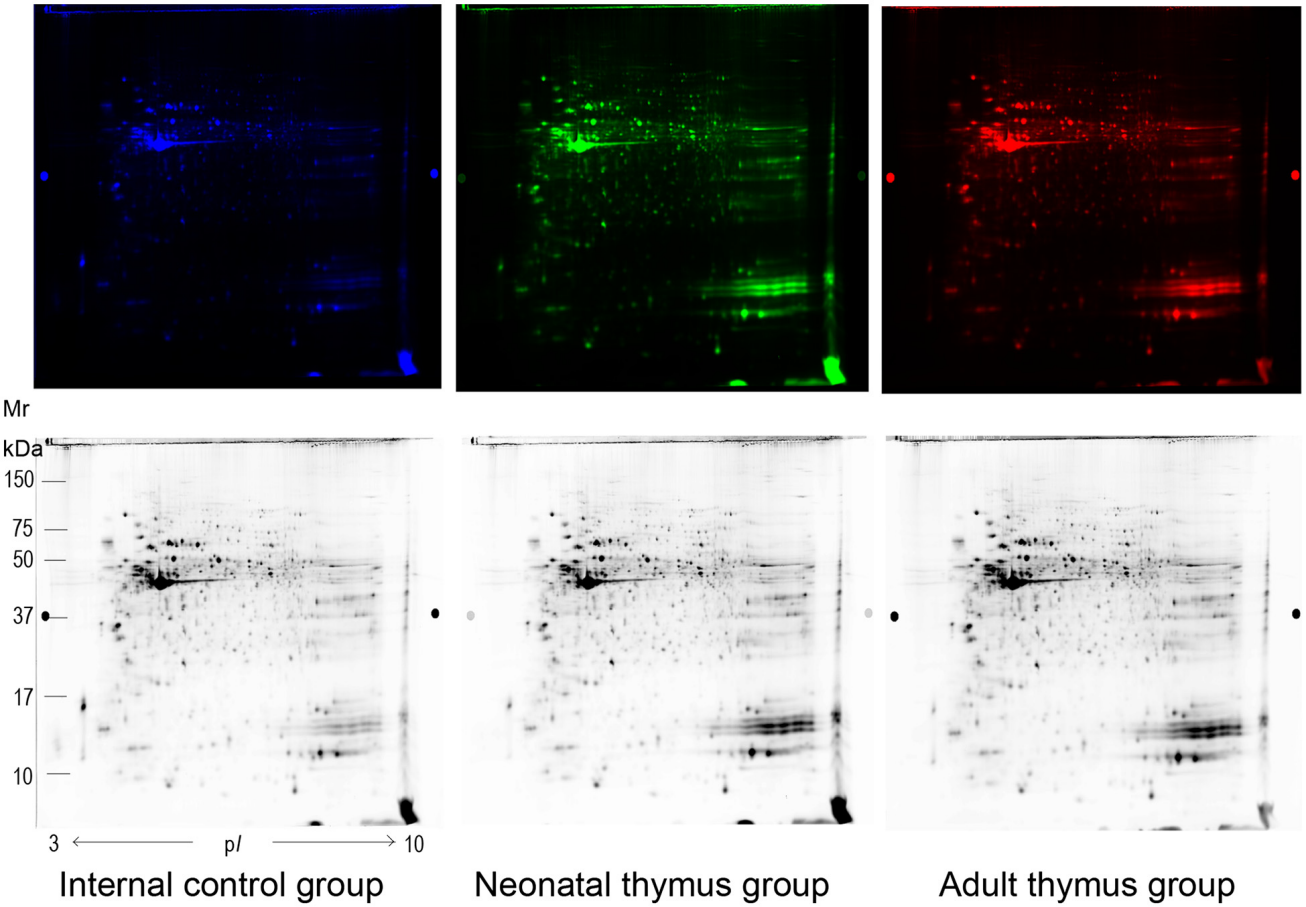
**Example of 2-D DIGE spectra in neonatal thymus and adult thymus.** The neonatal thymus sample was labeled with Cy3 Dye (green spots) and the adult sample with Cy5 Dye (red spots). In all gels, internal standard was labeled with Cy2 Dye.

Neonatal (labeled with Cy3 or Cy5) and adult (labeled with Cy3 or Cy5) spots were normalized to an internal standard (labeled with Cy2) containing equal amount of each protein extract used in the experiment. Differences in the two stages of thymus development are reflected in the proteomic profiles of the thymus by DIGE technology. A representative gel image is demonstrated from each group (Figure 1). Protein spots on the gel were clear and the majority of spots located at the region of pH 4–8 and relative molecular weight of 20–100 kDa. DeCyder 2-D difference analysis software was used to analyze and match 2-D images of the two groups. Overall, the average number of protein spots was 2274, and approximately 1406 proteins were matched between the two groups. Among them, we detected 317 spots with an increased level in the neonatal thymus and 194 spots with a higher representation in the adult thymus. 111 proteins of interest with the difference over 1.5-fold were identified by MALDI-TOF MS (Additional file 1: Table S1) and partial lists of them are indicated (Table 1).

**Table 1:** Partial lists of differentially regulated proteins in neonatal thymus group identified by MS^*^

**Accession No.**	**Protein name**	**Gene name**	**pI/MW**	**Mass matched**	**Protein covered (%)**	**Mascot score**	***t****-***test value**	**Fold change**
TENA_MOUSE	Tenascin	Tnc	4.77/237	11/22	8	70	0.00019	2.03↑
ACTN4_MOUSE	Actinin-4	Actn4	5.25/105	16/42	21	93	0.000054	1.74↑
TRAP1_MOUSE	Heat shock protein 75 kDa	Trap1	6.25/81	22/35	35	212	0.0000037	1.54↑
GRP75_MOUSE	Stress-70 protein	Hspa9	5.91/74	19/29	29	166	0.0000022	1.65↑
CH60_MOUSE	60 kDa heat shock protein	Hspd1	5.91/61	13/25	32	129	0.00012	1.90↑
ATPB_MOUSE	ATP synthase subunit beta	Atp5b	5.19/56	21/24	58	253	0.0000023	2.45↑
SEPT7_MOUSE	Septin-7	Sept7	8.73/51	4/16	12	103	0.000071	2.72↑
ACTB_MOUSE	Beta-actin	Actb	5.29/42	15/45	42	109	0.000075	2.20↑
STML2_MOUSE	Stomatin-like protein 2	Stoml2	8.95/38	14/24	45	165	0.00000052	2.70↑
PSME1_MOUSE	Proteasome activator complex subunit 1	Psme1	5.73/29	13/28	45	123	0.000015	3.57↓
COF1_MOUSE	Cofilin-1	Cfl1	8.22/19	7/9	34	96	0.00000032	9.36↓
COTL1_MOUSE	Coactosin-like protein	Cotl1	5.28/16	14/29	76	213	0.0000072	2.28↓

^*^CyDye images were analyzed by BVA and spots that showed statistically significant differences (*p*<0.05) in intensity between the neonatal and the adult thymus groups are listed. pI: calculated isoelectric point; MW: nominal molecular weight;↑: up-regulated; ↓: down-regulated.

### Ontogenic classification

**Figure 2:**
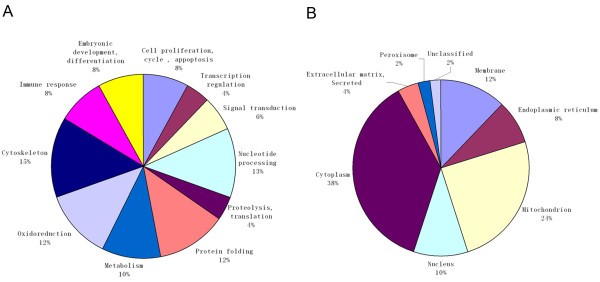
**Classification of proteins by gene ontology according to their biological function (A) and subcellular localization (B).** Assignments were made on the basis of information provided from the Swiss-Prot database.

The identified proteins with significant differential displays between the neonatal and the adult thymus group were clustered into categories according to their biological function and subcellular localization (Figure 2). Functional classification of identified proteins were grouped into 11 categories, including cell proliferation, cycle and apoptosis (8%), transcription regulation (4%), signal transduction (6%), nucleotide processing (13%), proteolysis and translation (4%), protein folding (12%), metabolism (10%), oxidoreduction (12%), cytoskeleton (15%), immune response (8%), and embryonic development (8%). According to the subcellular location, these proteins were classified into eight categories, including membrane (12%), endoplasmic reticulum (8%), mitochondrion (24%), nucleus (10%), cytoplasm (38%), extracellular matrix and secreted (4%), peroxisome (2%) and undefined (2%).

### Protein data mining

**Figure 3:**
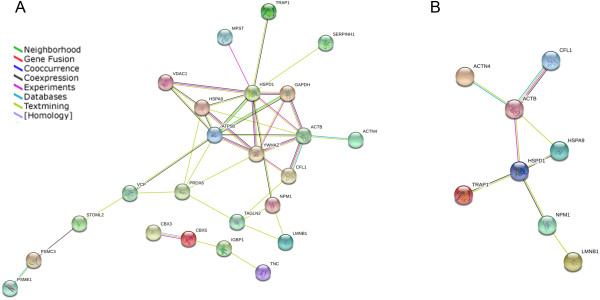
**The simulated functional network of proteins that were differentially displayed in the STRING database.** (**A**) Related networks and interactions between the identified proteins were shown. (**B**) Tight interactions centered on cytoskeletal proteins and chaperones.

The web-tool STRING is a database and web resource integrating information from numerous sources, including both physical and functional interactions [9]. Nodes represent the proteins and lines with different colors between nodes indicate different protein-protein interaction modes. Each interaction between nodes is supported from literatures. In this study, STRING identified the major networks comprised of cellular function and maintenance, cellular assembly, organization and metabolism, and it also showed the major interactions between the proteins which were differentially expressed (Figure 3).

### Validation of the differential protein displays by real time PCR and Western blotting

**Figure 4:**
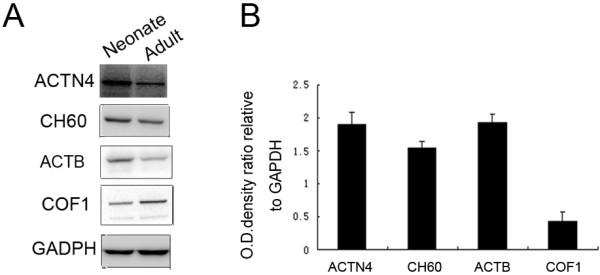
**Validation of differentially displayed proteins between the neonatal and adult thymus.** (**A**) 60 μg of protein extracts was used for protein detection. There were more abundant ACTN4, CH60 and ACTB expression, while less COF1 expression in neonates. GADPH was used as a normalization control. (**B**) A summary of densitometric analysis of Western blotting was shown as mean±SE calculated from three experiments. The results were normalized to individual GAPDH expression.

In order to confirm the changes described, experiments were next performed to validate the proteins differently display between the neonatal thymus and the adult. We selected 12 proteins differentially regulated at the two stages and quantified their mRNA expression by quantitative RT-PCR. As shown in Table 2, quantitative RT-PCR identified similar levels of mRNA regulation for these genes, indicating transcriptionally regulated expression of these proteins. We also analyzed samples by Western blotting using specific antibodies, which were directed against ACTN4, CH60, ACTB and COF1. The results obtained from Western blot were compatible with the intensities of the corresponding spots observed in 2-D gels (Figure 4). The results suggest that a proteomic differential display model is applicable to comparing.

**Table 2:** Differences in mRNA expression levels of the proteins differentially expressed in neonatal versus adult thymus

**Gene name**	**mRNA ratio (Neonate/Adult)**
Tnc	1.42
Atp5b	1.63
Actn4	1.78
Actb	1.71
Hspd1	1.48
Hspa9	2.23
Trap1	1.55
Sept7	1.89
Stoml2	1.60
Cfl1	0.76
Cotl1	0.68
Psme1	0.61

## Discussion

Proteomics studies, including 2-DE, MS, and bioinformatics tools, facilitate the direct understanding of the mechanism of the physiological or pathological process. A major critical issue of 2-DE involves reproducibility problems, owing to gel-to-gel and operator-to-operator variations [10,11]. To eliminate these technical limitations, investigators propose a complete workflow for DIGE image analysis and comparison in which cut-off values are carefully determined before extracting spots of interest according to the experimental conditions and reproducibility of the images analyzed. A detailed statistical analysis is also proposed on the identified spots using standard statistical tests [4,12].

Comparative proteomic analysis of proteins in mammalian organ at one point of time may accelerate pre- and clinical development of more specific diagnostic and prognostic disease markers and new, more selective therapeutic interventions [3-5,13]. Our study compared proteins in thymus from mice aged 1 day (neonates) with mice aged 60 days (adults) to gain insights into the proteins involved in the postnatal development of the thymus and the immune system. We found that there were many differences in the proteins expressed in adult and neonatal thymus, with approximately 511 differentially expressed proteins by more than 1.5-fold. Among them, 111 proteins were identified by MALDI-TOF MS. These proteins participate in the cell proliferation, cycle and apoptosis, transcription regulation, signal transduction, nucleotide processing, proteolysis and translation, protein folding, metabolism, oxidoreduction, cytoskeleton, immune response, and embryonic development.

According to our data, the proteins involved in structuring actin cytoskeleton have emerged from this study. Actb (Beta-actin), Actn4 (Actinin-4) and Sept7 (Septin-7) were found more expressed in the neonatal thymus compared to adult; Cfl1 (Cofilin-1) and Cotl1 (Coactosin-like protein) were found less expressed in the neonatal thymus. These proteins play a role in regulating the actin cytoskeleton, including filament polymerization and depolymerization. Increasing knowledge shows that the dynamic actin cytoskeleton, consisting of actin isoforms and their binding proteins, is essential for all developmental processes and the viability of the adult organism [14]. These functions are attributed to the ability of actin to form filaments that can rapidly assemble and disassemble according to the needs of the cell [15].

Actb is a highly conserved actin isoform ubiquitously expressed in vertebrates and mice hypomorphic for Actb die of uncharacterized defects during development [16,17]. Actn4 is an actin-binding protein and has been reported to crosslink actin, regulate actin cytoskeleton and enhance cell mobility [18]. Sept7, another cytoskeletal component, can assemble on the T-cell cortex and be enriched in filaments for efficient motion of motile T cells [19]. It is demonstrated that actin cytoskeleton and TCR signaling complexes are tightly integrated in T cells. Various cytoskeletal elements are crucial for the fine-tuning of T cell signaling and the immunological synapse (IS), while T cell activation induces the organization of both microtubules and actin cytoskeleton [20,21]. Thus these up-regulated cytoskeletal proteins assemble and contribute to T-cell activation and the IS formation in neonatal murine thymus. Additionally, we found Cfl1 and Cotl1 were down-regulated in the neonatal mice. It is reported that decreased Cfl1 expression is important for early mouse embryo development, and Cotl1 is associated with autoimmune disorders [22-24]. Both proteins belong to the actin depolymerizaition factor family and interact with actins and filaments to function primarily in promoting depolymerization [24,25]. Therefore, homeostasis of actin dynamics is important for the postnatal development of mice thymus.

The mitochondrial protein Stoml2 (Stomatin-like protein 2) was found more expressed in neonatal mice. It has been suggested that Stoml2 expression is dramatically up-regulated during T cell activation, and this increases T cell function and mitochondrial biogenesis, ultimately leading to resistance to apoptosis [26]. Interestingly, it is reported that Stoml2 is also involved in the organization of the peripheral cytoskeleton and acts as a functional bridge between TCR signalosomes and the cytoskeleton and cellular organelles [27].

These differentially expressed proteins involved in cytoskeleton household in neonatal compared to adult thymus are crucial for sustaining T cell activation and regulating cytoskeleton rearrangements. The regulation of cytoskeleton reorganization by T cell activation, and conversely, the control of T cell activation by cytoskeleton can be areas of active investigation. The disordered expression of these cytoskeleton proteins in neonatal thymus may be responsible for immune system diseases. Physiological variations occurring in the course of thymus maturation is a mandatory step to delineate the pathological mechanisms for the immune diseases which is related to cytoskeleton disruption. Accordingly, the cytoskeleton and its proteins greatly contribute to thymus maturation in neonatal mice, and cytoskeletal proteins can be potential targets for immunomodulation. Dysfunction of the cytoskeleton proteins in the development of thymus could lead to serious health consequences, including immunodeficiency or autoimmunity. Further investigation will be needed, and gene knockout or small molecular inhibitors that target these genes may be useful for understanding their effects in immune development.

Among the up-regulated proteins in neonatal mice, we also identified some heat shock proteins (HSPs) including Hspd1 (60 kDa heat shock protein), Trap1 (Heat shock protein 75 kDa) and Hspa9 (Stress-70 protein). These proteins represent a set of highly conserved molecular chaperones that serve by folding newly synthesized proteins, disassembling unstable proteins, and assisting in the transportation of proteins within the cell [28]. They enable cells to survive adverse environmental conditions, and their absence damages the embryonic development in mice [29,30]. Christensen *et al.* show that homozygosity for the null allele of *Hspd1* causes early embryonic lethality, while heterozygosity for the inactivated allele permits embryonic development and postnatal survival [30]. It is worth noting that eukaryotic and prokaryotic HSPs have high sequence homology and HSPs could act as potentially dangerous autoantigens, which adds to the evidence that neonatal mice are more susceptible to autoimmune disease than adult mice [31]. In addition, Npm1 (Nucleophosmin) was found highly expressed in neonatal thymus. Npm1 is a nuclear chaperone involved in chromatin remodeling during embryonic development and plays important roles in the regulation of cell proliferation and anti-apoptosis [32,33]. Loss of Npm1 impairs embryonic development and leads to premature cellular senescence and genomic instability [34]. Regarding these up-regulated chaperones above identified in neonatal thymus, it indicates their important roles during neonatal period.

Besides the proteins related with cytoskeleton and chaperones, other differentially expressed proteins were also identified. For instance, Tnc (Tenascin) is a glycoprotein of the extracellular matrix, which is involved in lymphocyte differentiation and migration. It is demonstrated that Tnc is expressed by epithelial cells early during embryonic development of the thymus [35,36]. It can support the tethering and rolling of lymphocytes, which would be used by lymphocytes migrating through secondary lymphoid organs [37]. Newborn mice are considered lymphopenic and the number of cells in the periphery is gradually increased by the constant output of newly exported T cells from the thymus [38,39]. Tnc is up-regulated in neonatal murine thymus, suggesting its important role in lymphocytic migration.

In addition, we also identified some proteins up-regulated in adult thymus. For example, Psme1 (Proteasome activator complex subunit 1) is implicated in immunoproteasome assembly and required for efficient antigen processing. Immune proteasomes in thymus are involved in processing of self-antigen, which are presented by MHC class I molecules for rejection of autoreactive thymocytes in adults [40,41]. They are present in adult thymus and responsible for negative selection of thymocytes through apoptosis. It is demonstrated that dexamethasone-induced thymocyte apoptosis is mediated by proteasomes, and lactacystin can also regulate apoptotic signaling as a proteasome-specific peptide inhibitor in the process of thymocyte apoptosis [42]. These implicate that such chemicals via regulating proteasome could be employed during the development, maturation or involution of thymus.

Bioinformatics analysis was performed to classify identified proteins in neonates based on biological function and subcellular localization. It links the identified proteins to protein folding, metabolism, oxidoreduction, cytoskeleton, immune response, embryonic development and so on. Some findings reported that neonates exhibited aberrant immune responses when compared to adults, resulting in increased susceptibility to infection and autoimmune disease [31,43]. Our results indicated the associated categories of proteins possibly involved in immaturity of neonatal for immune development and their potential role in neonates.

Recently the functional connectivity within a proteome becomes more and more important. As various protein complexes, transient interactions and functional pathways are all context-dependent, we further investigated the interaction networks between these proteins by the STRING web-tool. Although it represents the union of all possible protein-protein links, STRING imports protein association knowledge not only from databases of physical interactions, but also from databases of curated biological pathway knowledge [9]. The potential gene and protein interactions indicated in the study may enable prioritization of genes of interest. STRING results shows that those cytoskeletal proteins and chaperones are parts of the network that links the differentially expressed proteins. Most identified proteins are connected by the two kinds of proteins. Observing their own interaction network, we also found that they clustered in a tight interaction network centered on ACTB, HSPA9 and HSPD1. It is known that the cytoskeleton is incomplete without its associated proteins, which include chaperones that appear to protect the cytoskeleton in circumstances where cytoskeletal homeostasis is affected. The interplay between the chaperone and actin cytoskeleton also indicates that chaperones are not only limited to solve abnormal situations, but they also have an active participation during the normal differentiation process of the cell and are key factors for structural and functional rearrangements. For example, it has been reported that Prefoldin, a hexameric chaperone which facilitates posttranslational folding of actins and other cytoskeletal proteins, is required for lymphocyte development and function [44]. Deficiency in Prefoldin would cause lymphopoiesis defects, including dramatic reductions in immature CD4^+^ CD8^+^ double-positive T cells in thymus, and the phenotype was consistent with an actin-folding defect. Therefore, chaperones interact closely with the cytoskeleton network in the process of thymus maturation, and the additional experiments will be needed to identify more protein members and clarify the interaction and function.

In conclusion, we have identified unique differentially displayed proteins focusing on a comparison of immune related proteomes between neonatal thymus and adult thymus. The demonstrated molecular changes are relevant for understanding thymus development as well as neonatal immune function. Further studies will be required to describe in detail the role of the identified proteins in thymus maturation and in the specific functions of neonatal thymus.

## Materials and methods

### Animals

Eight different BALB/c mice were used in the study: four mice aged of 1 day and four mice aged of 60 days. They were kept under 12:12 h cycle of light with *ad libitum* access to food and drink. Mice were killed in accordance with Institutional Animal Care and Use Committee of China Medical University guidelines and thymuses were quickly dissected and frozen under liquid nitrogen. One whole-thymus tissue from each mouse was run on gels and each gel containing a pooled sample of the eight mice was run in parallel. The pooled sample was set as the internal pool, containing equal amount of each protein extract used in the experiment.

### Sample preparation and labelling

For protein solubilization, 1 mg lyophilized thymus was suspended in 400 μl rehydration buffer, i.e., 8 M urea, 2 M thiourea, 40 mM Tris, 4% CHAPS, 65 mM DTT, 2% IPG buffer and 1% protease inhibitor cocktail. The protein extracts were prepared for 2-DE by using 2-D clean-up kit (GE Healthcare) following manufacturer instructions. Precipitated proteins were resuspended in rehydration buffer and finally quantified using 2-D Quant Kit (GE Healthcare). The pH was adjusted to 8.5 by 100 mM NaOH and 50 μg of protein in either group was labeled with 400 pmol of either Cy3 or Cy5 dyes (GE Healthcare). A 50 μg protein mix, containing equal amount of each protein extract was labeled with 400 pmol of Cy2 dye as the internal standard sample. Labeled samples were immediately subjected to IPG strips (24 cm, pH 3–10, NL) and 900 μg of total protein was mixed in the rehydration buffer for preparative isoelectric focusing (IEF).

### 2-DE

IEF was performed using a step-wise voltage ramp by IPGphor IIIsystem (GE Healthcare): 30 V for 12 h, 300 V for 3 h, linear ramping from 300 V to 1,000 V for 6h and from 1,000 V to 8,000 V for 3 h, and finally 8,000 V for 7 h. Once IEF was completed, the strips were equilibrated in equilibration buffer (75 mM Tris–HCl, pH 8.8, 6 M urea, 30% glycerol, 2% SDS and 1% DTT) for 15 min, followed by the same buffer containing 2.5% iodoacetamide instead of DTT for another 15 min. The second dimension was performed using 12.5% SDS-PAGE gel (260×200×1 mm^3^) at 1 W constant power per gel by Ettan DALTsix (GE Healthcare).

### Image analysis

The gel was placed in the Typhoon 9400 Multi Scanner (GE Healthcare). Cy2, Cy3 and Cy5 fluorescence-labeled images were scanned at 488/520, 532/580 and 633/670 nm wavelength pairs, respectively. Quantitative differential expression analysis was performed by DeCyder 6.5 sftware (GE Healthcare). Scanned images of fluorescenty labeled proteins were sequentially analyzed by differential in-gel analysis (DIA-module) during which the Cy5:Cy2 and Cy3:Cy2 normalization of protein spot was performed. The Log abundance ratios of each protein spot were then compared between neonatal and adult thymus from all gels by Biological Variation Analysis (BVA-module). Due to an intrinsic variability associated to the mouse peculiarities, we chose a stringent criterion: (i) a change of expression of at least 1.5-fold, (ii) *t*-test value (*p*<0.05), and (iii) the identification of the spots in the four experimental replicates.

### Mass spectrometry identification and bioinformatics analysis

The differentially expressed protein spots were cut to reduction, alkylation, digestion, extraction, spot targeting and desalting. The sample plate was placed into MALDI-TOF mass spectrometer (Bruker Daltonics) for mass spectrometry (MS) analysis to obtain peptide mass fingerprinting (PMF). MS spectra were analyzed using the software flexAnalysis version 3.0 (Bruker Daltonics). Protein identification of peptide fragments was performed using MASCOT software (http://www.matrixscience.com) against Swiss-Prot database (Swiss Institute of Bioinformatics). Carbamidomethylation for cysteine, oxidation for methionine and other variants were also taken into consideration. Probability based on Mowse score >58 suggests a significant match and accurate identification of the protein.

### Interaction network

Functional partnerships between proteins are the fundamental of cell working. A proteome-scale interaction network of the differentially expressed proteins that identified in the present search was derived from the STRING database (http://string-db.org) [9].

### Quantitative real-time RT-PCR

Isolation of total RNA was carried out with the TRIzol (Invitrogen) according to the manufacturer’s protocol. One microgram of total RNA was reverse transcribed to cDNA in a total volume of 20 μl system using a RT reaction kit (Promega). Real-time PCR was performed using the Express SYBR greener qPCR supermix Universal Kit (Invitrogen) on a Rotor-gene 6000 system (QIAGEN). The 25-μl PCR mixture contained 2 μl reverse-transcribed product 12.5 μl SYBR Green supermix, 8.5 μl RNase-free water, 1 μl forward, and 1 μl reverse primers (Table 3). The reaction was incubated in a 72-well optical plate by 45 amplification cycles of 94°C for 5 s, 58°C for 20 s, and 72°C 30 s. Each sample was analyzed in triplicate and repeated three times. Gene expression levels were calculated relative to the housekeeping gene Gapdh.

**Table 3:** Primers used for RT-PCR

**Gene name**	**Primer sequence (5'-3')**	**Amplicon size (bp)**
Tnc	fwd: AGCCACCCGCTACTACAT rev: CTGCACCTGAACGACAAA	193
Atp5b	fwd: AGATTCTGGTGACTGGGATA rev: TGGCGACATTGTTGATTAG	132
Actn4	fwd: GTTTGCCTAAGCCAGAGC rev: ATCATTCCCAGGGTCATC	154
Actb	fwd: ATCGTGCGTGACATCAAA rev: AGAAGGAAGGCTGGAAAA	178
Hspd1	fwd: GGGGAAGTCCCAAAGTAA rev: CCTTGGCAATAGATCGTG	174
Hspa9	fwd: CAAAGGTCCTGGAGAATG rev: CAATAAGACGCTTAGTAGCA	150
Trap1	fwd: AGACGGACGCACCACTCA rev: CAGCCACTTGGGCAGGAT	158
Sept7	fwd: GTGAATCTGGACTGGGAAAG rev: CAGCAGCAACTGAACACCAC	158
Stoml2	fwd: GGGCTCTGACTCAACATAAT rev: GATTGGAGGGCAGTAGCA	117
Cfl1	fwd: TGCCGCTATGCACTCTAT rev: GGTCCTTGACCTCCTCGT	199
Cotl1	fwd: AAGTTTGCCCTCATCACA rev: ACTGAGCGTCGTAGTTGG	199
Psme1	fwd: AGGAGGAGCGGAAGAAGC rev: AACCAGGTAGTGACCAGATTGA	184
Gapdh	fwd: CCTTCCGTGTTCCTACCC rev: AAGTCGCAGGAGACAACC	163

### Western blot

To determine the expression of protein, tissue extracts were prepared from 1×10^6^ cells in lysis buffer (20 mM Tris pH7.4, 250 mM sodium chloride, 0.1%TritonX-100, 2 mM EDTA, 10 μg/ml leupeptin, 10 μg/ml aprotinin, 0.5 mM phenylmethylsulfonyl fluoride, 4 mM sodium orthovanadate and 1 mM DTT), and 60μg of the protein was resolved on 12% SDS-polyacrylamide gels. After electrophoresis, the proteins were eletrotransferred to nitrocellulose filters, the membrane (Amersham) was blocked with 5% nonfat dry milk in TBS-T (20 mM Tris, pH 7.6, 137 mM NaCl, 0.05% Tween-20) for 3 h at room temperature, and the proteins were probed with specific antibodies—ACTN4, CH60, ACTB and COF1 (Cell Signaling) and detected by chemiluminescence (Amersham). To assaure equal loading, gels were stripped and reprobed with antibodies against GAPDH (Shanghai Kangchen).

## Competing interests

The authors declare that they have no competing interests.

## Authors’ contributions

XC carried out experiments and data analysis, and composed the draft. WH carried out data acquisition and interpretation. YQ, YC and SD contributed to the proteomic analysis. SY and RC participated in supervision of the study. YJ contributed to the project idea and obtained grant funding. All authors have read and approved the final manuscript.

## Supplementary Material

Additional file 1: Table S1 Lists of differentially regulated proteins in neonatal thymus group identified by MS^*^. Click here for file
