# Supplementary material for: Proteomics identifies differentially expressed proteins in neonatal murine thymus compared with adults
Source: Proteome Sci. 2012 Nov 8;10:65. doi: 10.1186/1477-5956-10-65 (PMC3583686; doi:10.1186/1477-5956-10-65)
Supplement: Additional file 1: Table S1 — Lists of differentially regulated proteins in neonatal thymus group identified by MS*. [file 1477-5956-10-65-S1.doc]

| **Supplementary Table 1 Lists of differentially regulated proteins in neonatal thymus group identified by MS^*^** | | | | | | | |
| --- | --- | --- | --- | --- | --- | --- | --- |
| **Accession No.** | **Protein name** | **Gene name** | **pI/MW** | **Mass matched** | **Protein covered (%)** | **Mascot score** | **Fold change** |
| TENA_MOUSE | Tenascin | Tnc | 4.77/237 | 11/22 | 8 | 70 | 2.03↑ |
| CPSM_MOUSE | Carbamoyl-phosphate synthase | Cps1 | 6.30/166 | 11/15 | 9 | 86 | 1.63↑ |
| DNLI4_MOUSE | DNA ligase 4 | Lig4 | 8.17/105 | 6/14 | 12 | 58 | 1.59↑ |
| ACTN4_MOUSE | Actinin-4 | Actn4 | 5.25/105 | 16/42 | 21 | 93 | 1.74↑ |
| MVP_MOUSE | Major vault protein | Mvp | 5.34/100 | 16/37 | 18 | 122 | 1.92↑ |
| MCM6_MOUSE | DNA replication licensing factor MCM6 | Mcm6 | 5.32/94 | 4/9 | 7 | 119 | 1.75↑ |
| TERA_MOUSE | Transitional endoplasmic reticulum ATPase | Vcp | 5.14/90 | 11/20 | 18 | 94 | 1.51↑ |
| IMMT_MOUSE | Mitochondrial inner membrane protein | Immt | 6.18/84 | 19/25 | 22 | 195 | 1.85↑ |
| TRAP1_MOUSE | Heat shock protein 75 kDa | Trap1 | 6.25/81 | 22/35 | 35 | 212 | 1.54↑ |
| CAPR1_MOUSE | Caprin-1 | Caprin1 | 5.14/78 | 10/24 | 18 | 93 | 1.61↑ |
| FUBP2_MOUSE | Far upstream element-binding protein 2 | Khsrp | 6.90/77 | 17/25 | 31 | 192 | 1.62↑ |
| GUAA_MOUSE | GMP synthase | Gmps | 6.29/77 | 13/36 | 22 | 99 | 1.55↑ |
| NUCL_MOUSE | Nucleolin | Ncl | 4.69/77 | 4/11 | 7 | 185 | 1.78↑ |
| GRP75_MOUSE | Stress-70 protein | Hspa9 | 5.91/74 | 19/29 | 29 | 166 | 1.65↑ |
| DHSA_MOUSE | Succinate dehydrogenase | Sdha | 7.06/74 | 13/36 | 30 | 107 | 1.61↑ |
| AMPB_MOUSE | Aminopeptidase B | Rnpep | 5.51/73 | 9/26 | 17 | 75 | 1.53↑ |
| PLST_MOUSE | Plastin-3 | Pls3 | 5.41/71 | 13/22 | 25 | 131 | 1.64↑ |
| CALX_MOUSE | Calnexin | Canx | 4.50/68 | 8/24 | 16 | 66 | 1.81↑ |
| LMNB1_MOUSE | Lamin-B1 | Lmnb1 | 5.11/67 | 13/18 | 31 | 150 | 2.65↑ |
| HNRPL_MOUSE | Heterogeneous nuclear ribonucleoprotein L | Hnrnpl | 8.33/65 | 13/18 | 34 | 135 | 1.92↑ |
| ERG1_MOUSE | Squalene monooxygenase | Sqle | 8.67/64 | 7/24 | 16 | 67 | 1.84↑ |
| CH60_MOUSE | 60 kDa heat shock protein | Hspd1 | 5.91/61 | 13/25 | 32 | 129 | 1.9↑ |
| ATPA_MOUSE | ATP synthase subunit alpha | Atp5a1 | 9.22/60 | 19/38 | 33 | 145 | 1.66↑ |
| SYDC_MOUSE | Aspartate--tRNA ligase | Dars | 6.07/58 | 30/41 | 46 | 325 | 1.80↑ |
| PDIA1_MOUSE | Protein disulfide-isomerase | P4hb | 4.77/57 | 10/32 | 26 | 92 | 1.77↑ |
| ALDH2_MOUSE | Aldehyde dehydrogenase | Aldh2 | 7.53/57 | 9/22 | 17 | 286 | 1.59↑ |
| SCOT1_MOUSE | Succinyl-CoA:3-ketoacid coenzyme A transferase 1 | Oxct1 | 7.14/57 | 12/29 | 32 | 107 | 1.52↑ |
| ATPB_MOUSE | ATP synthase subunit beta | Atp5b | 5.19/56 | 20/37 | 54 | 253 | 2.45↑ |
| UGDH_MOUSE | UDP-glucose 6-dehydrogenase | Ugdh | 6.73/56 | 15/30 | 37 | 164 | 1.80↑ |
| K2C8_MOUSE | Keratin, type II cytoskeletal 8 | Krt8 | 5.70/55 | 18/37 | 37 | 135 | 1.88↑ |
| FUMH_MOUSE | Fumarate hydratase | Fh | 9.12/55 | 8/20 | 23 | 71 | 1.82↑ |
| SCMC1_MOUSE | Calcium-binding mitochondrial carrier protein SCaMC-1 | Slc25a24 | 6.00/54 | 9/32 | 24 | 67 | 1.61↑ |
| VIME_MOUSE | Vimentin | Vim | 5.06/54 | 20/31 | 50 | 210 | 1.75↑ |
| UFSP2_MOUSE | Ufm1-specific protease 2 | Ufsp2 | 6.28/53 | 10/25 | 29 | 101 | 1.74↑ |
| COR1A_MOUSE | Coronin-1A | Coro1a | 6.05/52 | 9/26 | 25 | 88 | 1.58↑ |
| SEPT7_MOUSE | Septin-7 | Sept7 | 8.73/51 | 4/16 | 12 | 103 | 2.72↑ |
| GDIB_MOUSE | Rab GDP dissociation inhibitor beta | Gdi2 | 6.11/51 | 15/38 | 41 | 142 | 1.65↑ |
| TBA1A_MOUSE | Tubulin alpha-1A chain | Tuba1a | 4.94/51 | 12/19 | 34 | 128 | 1.78↑ |
| NDUV1_MOUSE | NADH dehydrogenase | Ndufv1 | 8.51/51 | 6/36 | 19 | 95 | 1.63↑ |
| HNRPK_MOUSE | Heterogeneous nuclear ribonucleoprotein K | Hnrnpk | 5.39/51 | 10/15 | 32 | 135 | 1.78↑ |
| EFTU_MOUSE | Elongation factor Tu | Tufm | 7.26/50 | 16/26 | 40 | 172 | 1.69↑ |
| NUP50_MOUSE | Nuclear pore complex protein Nup50 | Nup50 | 5.94/50 | 7/26 | 27 | 65 | 1.79↑ |
| CSTF1_MOUSE | Cleavage stimulation factor subunit 1 | Cstf1 | 6.12/49 | 10/25 | 37 | 114 | 1.71↑ |
| HNRH1_MOUSE | Heterogeneous nuclear ribonucleoprotein H | Hnrnph1 | 5.89/49 | 10/27 | 32 | 102 | 1.71↑ |
| AATM_MOUSE | Aspartate aminotransferase | Got2 | 9.13/48 | 12/32 | 30 | 99 | 1.78↑ |
| WDR12_MOUSE | Ribosome biogenesis protein WDR12 | Wdr12 | 5.57/48 | 8/15 | 20 | 68 | 1.51↑ |
| K1C18_MOUSE | Keratin, type I cytoskeletal 18 | Krt18 | 5.22/48 | 12/31 | 39 | 125 | 1.87↑ |
| SERPH_MOUSE | Serpin H1 | Serpinh1 | 8.88/47 | 4/11 | 13 | 271 | 3.29↑ |
| ENOA_MOUSE | Alpha-enolase | Eno1 | 7.01/47 | 15/38 | 44 | 140 | 1.95↑ |
| PGK1_MOUSE | Phosphoglycerate kinase 1 | Pgk1 | 8.02/45 | 6/20 | 28 | 62 | 1.70↑ |
| TADBP_MOUSE | TAR DNA-binding protein 43 | Tardbp | 6.26/45 | 6/6 | 31 | 110 | 1.69↑ |
| THIL_MOUSE | Acetyl-CoA acetyltransferase | Acat1 | 8.71/45 | 7/21 | 20 | 109 | 1.95↑ |
| ACADS_MOUSE | Short-chain specific acyl-CoA dehydrogenase | Acads | 8.68/45 | 13/31 | 29 | 102 | 1.83↑ |
| ILEUA_MOUSE | Leukocyte elastase inhibitor A | Serpinb1a | 5.85/43 | 20/44 | 41 | 164 | 2.98↓ |
| DAZP1_MOUSE | DAZ-associated protein 1 | Dazap1 | 8.73/43 | 7/18 | 20 | 88 | 1.51↑ |
| ACTB_MOUSE | Beta-actin | Actb | 5.29/42 | 15/45 | 42 | 109 | 2.2↑ |
| DJB11_MOUSE | DnaJ homolog subfamily B member 11 | Dnajb11 | 5.81/41 | 13/36 | 39 | 118 | 1.53↑ |
| ALDOA_MOUSE | Fructose-bisphosphate aldolase A | Aldoa | 8.31/40 | 12/22 | 41 | 143 | 1.86↑ |
| ODPB_MOUSE | Pyruvate dehydrogenase E1 component subunit beta | Pdhb | 6.20/40 | 10/25 | 28 | 90 | 1.67↑ |
| GMPPB_MOUSE | Mannose-1-phosphate guanyltransferase beta | Gmppb | 6.27/40 | 6/20 | 26 | 62 | 1.59↑ |
| RM39_MOUSE | 39S ribosomal protein L39 | Mrpl39 | 8.09/39 | 17/28 | 48 | 164 | 1.66↑ |
| IPYR2_MOUSE | Inorganic pyrophosphatase 2 | Ppa2 | 6.51/39 | 9/26 | 24 | 69 | 1.71↑ |
| IGBP1_MOUSE | Immunoglobulin-binding protein 1 | Igbp1 | 5.81/39 | 9/20 | 30 | 78 | 2.55↓ |
| STML2_MOUSE | Stomatin-like protein 2 | Stoml2 | 8.95/38 | 14/24 | 45 | 165 | 2.7↑ |
| GBB2_MOUSE | Guanine nucleotide-binding protein G(I)/G(S)/G(T) subunit beta-2 | Gnb2 | 5.60/38 | 6/12 | 29 | 85 | 1.59↑ |
| GRAP2_MOUSE | GRB2-related adaptor protein 2 | Grap2 | 6.10/37 | 8/26 | 36 | 89 | 1.74↑ |
| ROA2_MOUSE | Heterogeneous nuclear ribonucleoproteins A2/B1 | Hnrnpa2b1 | 8.97/37 | 11/21 | 39 | 136 | 1.52↑ |
| CALU_MOUSE | Calumenin | Calu | 4.47/37 | 9/23 | 29 | 96 | 1.62↑ |
| PDLI1_MOUSE | PDZ and LIM domain protein 1 | Pdlim1 | 6.38/36 | 9/25 | 35 | 83 | 1.69↑ |
| GBLP_MOUSE | Guanine nucleotide-binding protein subunit beta-2-like 1 | Gnb2l1 | 7.60/36 | 12/23 | 55 | 155 | 1.74↑ |
| ECH1_MOUSE | Delta(3,5)-Delta(2,4)-dienoyl-CoA isomerase | Ech1 | 7.60/36 | 13/26 | 35 | 138 | 1.70↑ |
| HNRPC_MOUSE | Heterogeneous nuclear ribonucleoproteins C1/C2 | Hnrnpc | 4.92/34 | 8/20 | 24 | 190 | 1.91↑ |
| ROA1_MOUSE | Heterogeneous nuclear ribonucleoprotein A1 | Hnrnpa1 | 9.27/34 | 17/34 | 56 | 186 | 1.59↑ |
| NPM_MOUSE | Nucleophosmin | Npm1 | 4.62/33 | 6/17 | 14 | 114 | 1.53↑ |
| VDAC1_MOUSE | Voltage-dependent anion-selective channel protein 1 | VDAC1 | 8.55/33 | 9/25 | 35 | 122 | 1.87↑ |
| THTM_MOUSE | 3-mercaptopyruvate sulfurtransferase | Mpst | 6.11/33 | 13/27 | 45 | 179 | 7.04↑ |
| RSSA_MOUSE | 40S ribosomal protein SA | Rpsa | 4.79/33 | 9/19 | 42 | 124 | 1.51↑ |
| PNPH_MOUSE | Purine nucleoside phosphorylase | Pnp | 5.78/33 | 19/51 | 47 | 121 | 1.64↑ |
| PRDX4_MOUSE | Peroxiredoxin-4 | Prdx4 | 6.67/31 | 9/35 | 44 | 100 | 1.65↑ |
| PQBP1_MOUSE | Polyglutamine-binding protein 1 | Pqbp1 | 5.92/31 | 10/29 | 46 | 106 | 1.69↑ |
| APOA1_MOUSE | Apolipoprotein A-I | Apoa1 | 5.64/31 | 6/33 | 25 | 150 | 1.76↑ |
| NDUS3_MOUSE | NADH dehydrogenase [ubiquinone] iron-sulfur protein 3 | Ndufs3 | 6.67/30 | 15/23 | 40 | 238 | 1.85↑ |
| DNJC9_MOUSE | DnaJ homolog subfamily C member 9 | Dnajc9 | 5.58/30 | 12/30 | 45 | 135 | 1.74↑ |
| CWC15_MOUSE | Protein CWC15 homolog | Cwc15 | 5.55/27 | 7/22 | 18 | 85 | 1.90↑ |
| IDI1_MOUSE | Isopentenyl-diphosphate Delta-isomerase 1 | Idi1 | 5.79/27 | 12/35 | 56 | 129 | 1.81↑ |
| RN114_MOUSE | RING finger protein 114 | Rnf114 | 6.64/27 | 15/51 | 54 | 122 | 1.61↑ |
| MESD_MOUSE | LDLR chaperone MESD | Mesdc2 | 7.60/26 | 9/22 | 43 | 119 | 1.66↑ |
| CLCA_MOUSE | Clathrin light chain A | Clta | 4.45/26 | 5/23 | 15 | 171 | 1.64↑ |
| PRDX6_MOUSE | Peroxiredoxin-6 | PRDX6 | 5.71/25 | 9/16 | 44 | 135 | 7.65↑ |
| HPRT_MOUSE | Hypoxanthine-guanine phosphoribosyltransferase | Hprt1 | 6.21/25 | 6/26 | 32 | 63 | 1.88↑ |
| NDUS8_MOUSE | NADH dehydrogenase [ubiquinone] iron-sulfur protein 8 | Ndufs8 | 5.89/24 | 7/20 | 28 | 207 | 1.55↑ |
| RM12_MOUSE | 39S ribosomal protein L12 | Mrpl12 | 9.34/22 | 5/10 | 24 | 127 | 1.54↑ |
| CBX5_MOUSE | Chromobox protein homolog 5 | CBX5 | 5.71/22 | 3/7 | 21 | 155 | 2.14↑ |
| CBX3_MOUSE | Chromobox protein homolog 3 | CBX3 | 5.13/21 | 4/15 | 23 | 105 | 5.18↑ |
| FABP5_MOUSE | Fatty acid-binding protein | Fabp5 | 6.14/15 | 11/24 | 82 | 138 | 1.66↑ |
| ERH_MOUSE | Enhancer of rudimentary homolog | Erh | 5.63/12 | 5/13 | 38 | 110 | 1.80↑ |
| BAF_MOUSE | Barrier-to-autointegration factor | Banf1 | 5.79/10 | 5/21 | 62 | 148 | 1.91↑ |
| UBA1_MOUSE | Ubiquitin-like modifier-activating enzyme 1 | Uba1 | 5.49/119 | 15/26 | 16 | 130 | 1.68↓ |
| PRS6A_MOUSE | 26S protease regulatory subunit 6A | Psmc3 | 8.91/50 | 9/29 | 15 | 79 | 1.78↓ |
| PSD13_MOUSE | 26S proteasome non-ATPase regulatory subunit 13 | Psmd13 | 5.53/43 | 8/10 | 32 | 128 | 1.85↓ |
| G3P_MOUSE | Glyceraldehyde-3-phosphate dehydrogenase | Gapdh | 8.44/36 | 12/29 | 40 | 145 | 2.4↓ |
| PHB_MOUSE | Prohibitin | Phb | 5.57/30 | 12/45 | 58 | 121 | 1.72↓ |
| PSME1_MOUSE | Proteasome activator complex subunit 1 | Psme1 | 5.73/29 | 13/28 | 45 | 123 | 3.57↓ |
| 1433Z_MOUSE | 14-3-3 protein zeta/delta | Ywhaz | 4.73/28 | 14/21 | 36 | 141 | 5.41↓ |
| TAGL2_MOUSE | Transgelin-2 | Tagln2 | 8.39/23 | 22/37 | 66 | 224 | 2.05↓ |
| COF1_MOUSE | Cofilin-1 | Cfl1 | 8.22/19 | 7/9 | 34 | 96 | 9.36↓ |
| NDKB_MOUSE | Nucleoside diphosphate kinase B | Nme2 | 6.97/17 | 9/27 | 52 | 117 | 1.93↓ |
| COTL1_MOUSE | Coactosin-like protein | Cotl1 | 5.28/16 | 14/29 | 76 | 213 | 2.28↓ |
| CYB5_MOUSE | Cytochrome b5 | Cyb5a | 4.96/15 | 6/19 | 42 | 101 | 1.93↓ |
| QCR7_MOUSE | Cytochrome b-c1 complex subunit 7 | Uqcrb | 9.10/14 | 6/24 | 49 | 150 | 1.58↓ |
| CX6B1_MOUSE | Cytochrome c oxidase subunit 6B1 | Cox6b1 | 8.96/10 | 7/21 | 72 | 247 | 2.53↓ |
| ^*^CyDye images were analyzed by BVA and spots that showed statistically significant differences (*p*<0.05) in intensity between the neonatal and the adult thymus groups are listed. pI: calculated isoelectric point; MW: nominal molecular weight;↑: up-regulated; ↓: down-regulated. | | | | | | | |
